# Acute Peripheral Facial Paralysis Masquerading as Bell's Palsy Is the First Presentation of COVID-19 Infection

**DOI:** 10.1155/2023/4278146

**Published:** 2023-01-30

**Authors:** Tabtim Chongsuvivatwong, Panitta Mueanchoo, Praewchompoo Sathirapanya, Pornchai Sathirapanya

**Affiliations:** ^1^Department of Medicine, Hat Yai Hospital, Songkhla 90110, Thailand; ^2^Sikarin Hat Yai Hospital, Songkhla 90110, Thailand; ^3^Sabayoi Hospital, Songkhla 90210, Thailand; ^4^Department of Internal Medicine, Faculty of Medicine, Prince of Songkla University, Songkhla 90110, Thailand

## Abstract

Although Bell's palsy is a common diagnosis of acute isolated peripheral facial palsy (PFP), acute isolated PFP can be the first presentation of various illnesses, including COVID-19 disease. A female with a known history of well-controlled diabetes mellitus presented initially with acute isolated PFP mimicking Bell's palsy. A course of oral prednisolone was given to treat acute PFP. Severe fifth cervical radicular pain, which is unusual for Bell's palsy followed 3 days later. The COVID-19 infection was finally diagnosed by a real-time polymerase chain reaction (RT-PCR) test 15 days after facial paralysis when typical pulmonary infection symptoms developed. Oral favipiravir was given for the treatment of COVID-19 infection, to which the symptoms completely responded. The COVID-19 infection as a cause of acute isolated PFP should be added to the differential diagnosis of acute isolated PFP, albeit without typical pulmonary infection symptoms, particularly during the global pandemic of the infection.

## 1. Introduction

Bell's palsy or idiopathic facial palsy is the most common diagnosis of acute isolated peripheral facial palsy (PFP); however, other causative organisms and etiologies of PFP have been reported, such as herpes simplex, varicella zoster, *Epstein-Barr* virus, *Borrelia burgdorferi*, chronic otitis media, cholesteatoma, facial nerve schwannoma, and acoustic neuroma [1–3]. Acute isolated PFP as the first presentation before the typical respiratory and systemic inflammatory syndrome (SIS) of COVID-19 infection is rarely reported. A study reported that most of the COVID-19-associated PFP cases acquired PFP after the diagnosis of COVID-19 infection [4]. The current case report presents a COVID-19-infected patient who experienced acute isolated PFP substantially long before the onset of the typical pulmonary and systemic illnesses of COVID-19 disease.

## 2. Case Presentation

**Figure 1:**
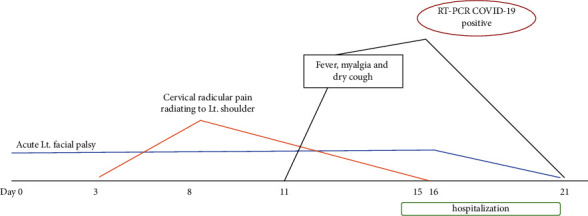
Illustration of clinical course of the reported patient.

**Figure 2:**
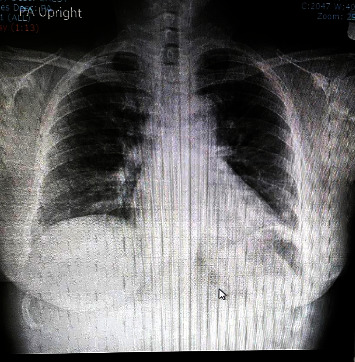
Chest radiography on admission.

A 57-year-old female with a history of diabetes mellitus but no diabetic complications noticed left peripheral facial paralysis when she woke up one morning in early April 2021. She had no associated fever, headache, or systemic or neurological disorders. Isolated left PFP of House–Brackmann scale (HBs) grade 4/6 was confirmed on the initial neurological evaluation at the outpatient department. Bell's palsy was diagnosed at the patient's first presentation. Hence, a 5-day course of 60 mg/d of oral prednisolone followed by a 10 mg/d tapering-off regimen was prescribed. Three days after the first presentation, she reported severe neuropathic pain on her left shoulder and upper arm following the sensory dermatome of the C5 spinal nerve root. The excruciating neuropathic pain needed a combination of antineuropathic pain and opioid drugs prescribed by a physician in the emergency department. No new abnormal neurological signs were noted at that time, so no blood tests or neurological imaging were done as well. The neuropathic pain progressed for the next 5 days and then gradually subsided while the PFP remained stable. On day 11 of the illness, the patient had high fever, dry cough, malaise, and severe muscle aches. She was hospitalized on day 15 of illness due to progressive pulmonary infection symptoms (Figure 1). On the admission date, her body temperature was 38.9°C., respiratory rate 26/min, pulse rate 98/min, and blood pressures 131/83 mmHg. Minimal fine crackle sound was auscultated from both basal lung fields. No cardiac murmur, hepatosplenomegaly, or lymphadenopathy was found. The only abnormal neurological sign found was the isolated PFP, which had recovered to HBs grade 2/6. The previous radiculopathic pain disappeared. A fundoscopic examination showed no papilledema and normal retinal vessels. Motor power, sensory functions, deep tendon reflexes, and cerebellar functions were all normal. No neck stiffness was found. Electrocardiography was unremarkable. However, a chest film showed a ground glass appearance on both basal lungs (Figure 2). Desaturation of arterial blood oxygen with PaO_2_ of 92% evaluated by a finger-tip oximeter was noted where a high-flow nasal canula was initiated. The laboratory results showed a complete blood count of leucopenia (4,700 cells/*μ*L), lymphopenia (1,020 cells/*μ*L), and thrombocytopenia (1.5 × 10^5^ cells/mm^3^). Fasting blood sugar was 132 mg/dL (70–100 mg/dL), HbA1C 7.1% (4.5–6.3%), blood urea nitrogen 11 (9.8–20.1 mg/dL), creatinine 0.7 (0.5–1.02 mg/dL), serum sodium 140.9 (136–145 mEq/L), potassium 4.22 (3.5–5.0 mEq/L), chloride 104.1 (98–107 mEq/L), and CO_2_ 24 (22–29 mEq/L). C-reactive protein was as high as 56.39 mg/L (<5 mg/L). Liver function tests, serum albumin, and serum globulin were all unremarkable. A real-time PCR done on nasopharyngeal swab specimen reported the presence of SARS-CoV-2 viral genome (cycle threshold (Ct) values of open reading frame (ORF)1ab and N genes were 30.40 and 26.11, respectively). Anti-HIV and anti-*Treponema pallidum* antibodies reported negative results, but herpes simplex virus (HSV) IgG and varicella zoster virus (VZV) IgG were positive, indicating the previous infections. Since Thailand is not an endemic area for Lyme disease, the serology test for *B. burgdorferi* was not available. The patient had a history of COVID-19 contact, which was revealed later when her family members were tested positive for RT-PCR of COVID-19. Subsequently, COVID-19 pneumonitis was diagnosed and a 10-day course of oral favipiravir, consisting of 1800 mg twice a day on the first day and 800 mg twice a day on the 9 succeeding days, was prescribed to the patient. During admission, the respiratory and systemic illness as well as facial paralysis gradually recovered to complete resolution in 3 weeks after admission.

## 3. Discussion

After the first outbreak of COVID-19 infection, originally known as the novel corona virus disease 2019, in Wuhan, China, in December 2019, it was declared a global health crisis by the WHO in February 2020 due to its high contagion and fatality rate. In Thailand, the first COVID-19 confirmed case was reported in a Chinese tourist in January 2020. Then, two succeeding limited outbreaks occurred in the year 2020. The third widespread outbreak of COVID-19 infection over the country began in early April 2021 with a rapidly increasing number of COVID-19 confirmed cases.

In this case report, acute isolated PFP was the first and sole presentation of COVID-19 infection. The typical respiratory tract symptoms of COVID-19 infection did not appear until 11 days after the onset of PFP. The first presentation with acute isolated PFP misled the diagnosis to Bell's palsy because it is the most common diagnosis of acute isolated PFP [1]. It is not unusual that Bell's palsy was the first diagnosis in this patient, and until typical pulmonary and systemic infection symptoms appeared, a COVID-19 infection was finally diagnosed. Usually, a diagnosis of Bell's palsy is primarily based on compatible clinical characteristics and the exclusion of other possible causes of acute isolated PFP [3]. A study by Lima et al. reported 8 cases of COVID-19-associated PFP in which PFP was the first presentation in 3 of them, while the remaining cases acquired PFP 2–10 days after the onset of respiratory symptoms [4]. Furthermore, PFP was usually associated with Guillain–Barre syndrome (GBS) in COVID-19 infection. Usually, most of the COVID-19-associated polyneuropathies, including GBS or cranial neuropathy, occur at a median time of 7 (−7 to 24) days in relation to the onset of respiratory illness [5]. Hence, it can be inferred that acute isolated PFP is less likely to be the sole presenting symptom of COVID-19 infection.

Although the definite pathogenesis of Bell's palsy has not been confirmed, facial nerve demyelination caused by immunologic reactions to some preceding infections, particularly Herpes virus infection, was suggested [6]. Moreover, microangiopathy of the vasa nervorum supplying the facial nerve caused by immune-mediated vasculitis is another possible pathogenic mechanism of Bell's palsy [6]. As it is considered a postinfectious facial neuropathy as mentioned, Bell's palsy typically presents as an isolated PFP without any associated systemic or neurological disorders. Only a few days of heralding auricular or mastoid pain, which is the sensory area innervated by the sensory branches of the trigeminal, facial, glossopharyngeal, vagus nerves, and the C2-3 spinal nerve roots could present in some cases of Bell's palsy [7]. The anastomosis between facial nerves and these cranial and cervical nerve roots is an explanation of the symptom. Hence, the C5 radiculopathic pain in association with acute PFP in this patient is unusual for a diagnosis of Bell's palsy.

The definite pathogenic mechanism of acute PFP in COVID-19 infection remains unclarified. The possible mechanisms include direct infection of the facial nerve by the SARS-CoV-2 virus, immunologic microangiopathy of the vasa nervorum supplying the facial nerve, or immune-mediated demyelination of the facial nerve like that of Bell's palsy. Since the SARS-CoV-2 viral genome were not detectable from the cerebrospinal fluid by real-time PCR in the patients presented with various neurological disorders including cranial or peripheral neuropathy, direct COVID-19 viral infection of nervous tissue was unlikely [4, 5, 8]. On the other hand, because SARS-CoV-2 virus induces intense immune reactions and inflammation by cytokine storms, immune-mediated facial neuropathy during COVID-19 infection is considered possible. The presentations of our case report are similar to those reported in the articles [4, 5]. The article by Ellul et al. also proposed that because acute PFP commonly develops shortly before or after the onset of pulmonary or systemic illness of COVID-19 infection, it could be considered a parainfection of COVID-19 infection [5]. Because there are no other identifiable causes to explain acute isolated PFP in this case report, it is possible that the acute PFP in our case was associated with COVID-19 infection by the timeline association and could be a parainfection of COVID-19 infection as mentioned in the article of Ellul et al. [5]. Since the serum HSV and VZV IgG were found in this patient, reactivation of these viruses causing acute PFP like Bell's palsy may be possible. However, the emerging severe and long duration of C5 radicular pain after the development of facial palsy are considered atypical for a diagnosis of Bell's palsy both the level of cervical root involved and the time of onset association. Moreover, facial neuropathy in conjunction with painful radiculopathy complicated by diabetes mellitus should be considered as well. Nevertheless, we think this is unlikely because the patient had been in good glycemic control and no macrovascular or microvascular complications related to diabetes mellitus presented. Even though there is no cerebrospinal fluid analysis or biomarker assay to confirm the potential diagnosis of parainfection related to COVID-19 infection, we consider that the acute isolated PFP described in this case report should be a parainfection symptom in association with COVID-19 infection.

The treatment of PFP associated with COVID-19 is similar to that of Bell's palsy based on the presumably shared pathogenesis of immune facial neuropathy [2]. Oral prednisolone was prescribed to the patient with a favorable outcome that the facial paralysis completely recovered within 3 weeks following the expected response time in cases of Bell's palsy generally.

## 4. Conclusions

During a COVID-19 pandemic, acute isolated PFP is possibly the first presentation of the infection. The etiologies of acute PFP other than Bell's palsy should be concerning, particularly when atypical related symptoms of Bell's palsy are concomitantly present. The occurrence of C5 radicular pain after the onset of PFP and the manifestation of COVID-19 pulmonary symptoms in this patient are very unusual for a diagnosis of Bell's palsy. From now on, it can be suggested that COVID-19 infection should be added to the differential diagnosis of acute isolated PFP.

## Data Availability

The data used to support the findings of this study are included within the article.

## References

[1] Hohman M. H., Hadlock T. A. (2014). Etiology, diagnosis, and management of facial palsy:2000 patients at a facial nerve center. *The Laryngoscope*.

[2] Masterson L., Vallis M., Quinlivan R., Prinsley P. (2015). Assessment and management of facial nerve palsy. *BMJ*.

[3] Fuller G., Morgan C. (2016). Bell’s palsy syndrome: mimics and chameleons. *Practical Neurology*.

[4] Lima M. A., Silva M. T. T., Soares C. N. (2020). Peripheral facial nerve palsy associated with COVID-19. *Journal of NeuroVirology*.

[5] Ellul M. A., Benjamin L., Singh B. (2020). Neurological associations of COVID-19. *The Lancet Neurology*.

[6] Eviston T. J., Croxson G. R., Kennedy P. G. E., Hadlock T., Krishnan A. V. (2015). Bell’s palsy: aetiology, clinical features and multidisciplinary care. *Journal of Neurology, Neurosurgery & Psychiatry*.

[7] Jaber J. J., Leonetti J. P., Lawrason A. E., Feustel P. J. (2008). Cervical spine causes for referred otalgia. *Otolaryngology - Head and Neck Surgery*.

[8] Paybast S., Emami A., Koosha M., Baghalha F. (2020). Novel coronavirus disease (COVID-19) and central nervous system complications: what neurologist need to know. *Acta Neurol Taiwan*.

